# 基于气相色谱-四极杆-飞行时间质谱高通量筛查芝麻油中54种替代型增塑剂

**DOI:** 10.3724/SP.J.1123.2023.08007

**Published:** 2023-11-08

**Authors:** Keyun CHEN, Xiang JU, Yanli WANG, Xiuli XU, Xiuqing LIANG, Haixia LI, Xia LI, Fangfang LI, Qiyan TIAN, Qianqian CHEN, Yanming LIU, Feng ZHANG

**Affiliations:** 1.山东省食品药品检验研究院, 国家市场监管重点实验室(肉及肉制品监管技术), 产业技术基础公共服务平台, 山东 济南 250101; 1. Shandong Institute for Food and Drug Control, Key Laboratory of Supervising Technology for Meat and Meat Products for State Market Regulation, Industrial Technology Foundation Public Service Platform, Jinan 250101, China; 2.中国检验检疫科学研究院, 北京 100123; 2. Chinese Academy of Inspection and Quarantine, Beijing 100123, China

**Keywords:** 气相色谱-四极杆飞行时间质谱, 替代型塑化剂, 高通量筛查, 芝麻油, gas chromatography-quadrupole time-of-flight mass spectrometry (GC-Q-TOF/MS), alternative plasticizers (APs), high-throughput screening, sesame oil

## Abstract

以气相色谱-四极杆-飞行时间质谱(GC-Q-TOF/MS)为检测手段,建立了芝麻油中54种替代型塑化剂(APs)的高通量快速筛查方法。样品经乙腈提取、PSA/Silica固相萃取柱净化后,经GC-Q-TOF/MS的全扫描模式采集质谱信息,利用构建的54种APs高分辨质谱数据库进行检索来实现广谱筛查、定性鉴别,并同时对5种目标物进行定量分析。比较了不同提取溶剂种类、不同净化方式对样品提取与净化的影响,通过优化气相色谱分离条件,优化筛查参数来提高筛查结果的准确性。结果表明,54种APs的筛查限(SDL)为0.01~0.02 mg/kg,定量限为0.02~0.04 mg/kg。利用该方法对市售的80批次芝麻油样品进行快速筛查分析,有5种化合物得到鉴定,对鉴定到的5种化合物进行基质匹配外标法定量分析。结果显示,5种APs在0.01~0.2 mg/L范围内线性关系良好,相关系数均大于0.99,芝麻油空白样品加标回收试验结果表明,5种APs的回收率为71.3%~97.8%,相对标准偏差(RSD)为0.4%~6.1%(*n*=6)。该方法快速、准确,灵敏度高,检测通量高,实现了芝麻油中54种替代型塑化剂的高效筛查、定性鉴别及阳性样品的定量分析,为食品中其他污染物的监测提供了参考。

## Abstract (en)

Restrictions on the use of phthalates have led to the wide use of alternative plasticizers (APs) such as organophosphate, adipate, citrate, and sebacate. However, because plasticizers combine with polymers in plastic products via unstable noncovalent bonds, they can easily migrate out of these products, causing environmental pollution. In particular, their migration out of food packaging, containers, and other food-contact materials and into food has raised great concerns. Toxicological studies have shown that APs contain potentially toxic substances that can affect endocrine functions and cause neurotoxicity, genotoxicity, and other adverse effects. Thus, their potential risks to food should not be underestimated. Sesame oil is a necessity in daily cooking. The results of risk monitoring in recent years have indicated that sesame oil often contains phthalates in excess of the standard limits. However, the potential risks of APs in sesame oil have not yet been reported. Some common detection methods for APs include gas chromatography-mass spectrometry, gas chromatography-triple quadrupole mass spectrometry, and liquid chromatography-triple quadrupole mass spectrometry. Unfortunately, these methods use low-resolution mass spectrometry and are limited by the resolution, scan rate, and analysis mode. Gas chromatography-quadrupole time-of-flight mass spectrometry (GC-Q-TOF/MS) has the advantages of high resolution, sensitivity, and analysis speed. In full-scan mode, GC-Q-TOF/MS can accurately collect the full-spectrum mass number of target compounds with low content levels in complex substrates, thereby realizing efficient screening and quantitative analysis. It shows outstanding advantages in the trace analysis of pesticide residues and pollutants. Furthermore, it features strong qualitative and high screening abilities. Establishment of a personal compound database and library (PCDL) addresses limitations in the number of compounds that can be measured and enables the rapid identification of targets without the use of standard products. In addition, increasing the number of targets for synchronous screening enables the retrospective analysis of new targets.

In this study, a method based on GC-Q-TOF/MS was developed for the determination of 54 APs in sesame oil. The samples were extracted with acetonitrile and purified using a PSA/silica solid-phase extraction column. The mass-spectral information of the samples was then collected by GC-Q-TOF/MS in full-scan mode, and the 54 APs were searched using an established high-resolution mass-spectrum database to simultaneously achieve the broad-spectrum screening, qualitative identification, and quantitative analysis of multiple targets. The effects of different extraction solvents and purification methods on sample extraction and purification were compared. The accuracy of the screening results was improved by optimizing the GC-separation conditions, quality-extraction window, retention-time deviation, and other screening parameters. The screening detection limits (SDLs) of the 54 APs ranged from 0.01 to 0.02 mg/kg; specifically, the SDL of 41 compounds was 0.01 mg/kg and that of 13 compounds were 0.02 mg/kg. The limits of quantification were in the range of 0.02-0.04 mg/kg. A total of 80 sesame-oil samples were rapidly screened using this method under optimal conditions. Five APs were identified from the 80 sesame-oil samples and quantitatively analyzed using the matrix-matched external-standard method. The results of this quantitative methodology showed that the five APs had good linear relationships in the range of 0.01-0.2 mg/L, with all correlation coefficients greater than 0.99. The accuracy and precision of the method were verified using a standard recovery test with blank sesame-oil samples. Under the three standard levels of 0.04, 0.08, and 0.2 mg/kg, the recoveries of the five APs ranged from 71.3% to 97.8%, and the relative standard deviations (RSDs) ranged from 0.4% to 6.1%(*n*=6). The developed method is fast, accurate, sensitive, and has high throughput. Thus, it can realize the efficient screening, qualitative identification, and quantitative analysis of the 54 APs in sesame oil and provides a potential solution for the monitoring of other contaminants in food.

近年来,随着邻苯二甲酸酯类物质被限制使用,替代型塑化剂(alternative plasticizers, APs)得到更广泛的使用。APs是指除邻苯二甲酸酯以外的塑化剂,主要包括有机磷酸酯、己二酸酯、柠檬酸酯、癸二酸酯及邻苯二甲酸酯外的苯二甲酸酯类化合物等。研究发现,APs与塑料制品中的聚合物是以不稳定的非共价键形式结合的,因此很容易从塑料制品中迁移出来,造成环境,特别是食品的污染,食品中的APs除了从环境吸收外,主要来自包装、容器等食品接触材料中的迁移^[1,2]^。然而毒理学研究表明,APs是重要的有潜在毒性的物质,比如己二酸二(2-乙基己基)酯(DEHA)、乙酰柠檬酸三丁酯(ATBC)和2,2,4-三甲基-1,3-戊二醇二异丁酸酯(TXIB)为内分泌干扰物,会导致性类固醇稳态功能障碍^[3]^;对苯二甲酸二(2-乙基)己酯会影响内分泌,与育龄妇女子宫肌瘤有关^[4,5]^;有机磷酸酯具有神经毒性、基因毒性等^[6,7]^,其在食品中的潜在风险不容轻视。

国内外相继出台法规限制食品包装材料中APs的使用,规定了其在食品中的特定迁移限量(SML),如欧盟法规(EU)No 10/2011《关于与食品接触的塑料材料和制品》、我国GB 9685-2016《食品接触材料及制品用添加剂使用标准》规定间苯二甲酸二甲酯、TXIB、DEHA等的SML分别为0.05、5、18 mg/kg。目前研究证实APs广泛存在于各种基质中,如环境(含空气、灰尘、污泥、海水沉积物)^[8⇓⇓⇓⇓⇓-14]^、食品(鱼、蛋、肉、牛奶、快餐食品、含乳饮料)^[15⇓⇓-18]^和生物基质(尿液、头发)^[19⇓-21]^。此外还涉及食品接触材料^[22⇓-24]^、医疗器械^[25,26]^、口罩^[27]^等样本。有研究报道在中国南京收集的食品样本中检出柠檬酸三丁酯,其最高含量为0.128 mg/kg^[28]^,此外,在日本、西班牙等肉类、蔬菜、谷物、清酒等食品中ATBC均有较高的检出浓度和检出频率,日本清酒中ATBC的含量高达7.30 mg/kg^[29,30]^。

芝麻油是居民日常烹饪的必需品,随着我国生活水平的提高,其消费量持续上升,已达40多万吨^[31]^。然而,从近年来我国的风险监测结果来看,芝麻油中常检测出邻苯二甲酸酯类物质超标^[32]^。对于芝麻油中APs的潜在风险尚未见报道,APs被认为是增塑剂行业的未来发展方向,掌握它们在芝麻油中的数据有利于制定监管措施,推动整个行业健康发展。

常见的APs检测方法主要有气相色谱-质谱法^[11,22,33]^、气相色谱-三重四极杆质谱法^[13,16]^及液相色谱-三重四极杆质谱法^[8,19]^等,这些方法均使用低分辨质谱,受到分辨率、扫描速率及分析模式的限制。当样品基质复杂时,目标物和干扰物不能有效区分,常出现假阳性而造成误判,在分析几百种目标物时,分组进行检测会降低分析速度,限制了扫描的化合物数量,无法真正实现高通量筛查,而且,定性分析必须依赖标准物质,增加了实验成本和工作量。气相色谱-四极杆-飞行时间质谱(GC-Q-TOF/MS)具有分辨率高、灵敏度好、分析速度快等优点,在全扫描模式下可对复杂基质中的低含量水平目标化合物进行精确质量数全谱采集,实现高效筛查和定量分析,在农药残留、污染物痕量分析中表现突出^[34,35]^。其定性能力强、筛查能力高,测定化合物不受数量限制,通过建立PCDL(personal compound database and library)数据库可实现不使用标准品对目标物进行快速鉴定,并能够对新目标物进行回溯性分析,增加了同步筛查的目标物数量。

本研究以芝麻油为研究对象,拟利用GC-Q-TOF/MS构建基于PCDL库的高通量筛查技术,实现54种高关注、高风险的APs的高效筛查及定量分析,为芝麻油中APs的风险评估和风险监测提供有力的技术支撑。

## 1 实验部分

### 1.1 仪器、试剂与材料

Agilent 8890气相色谱-7250四极杆/飞行时间质谱仪(美国安捷伦科技有限公司); SQP型电子天平(精度0.01 g,北京赛多利斯天平有限公司); MS3涡旋混合器(德国IKA公司); KQ-800DE超声波清洗机(昆山市超声仪器有限公司); N-EVAP116氮吹仪(美国Organomation公司); 3-18KS离心机(德国Sigma公司); HP-5MS色谱柱(30 m×250 μm×0.25 μm,美国安捷伦公司)。

乙腈、正己烷、二氯甲烷均为色谱纯,购自德国默克有限公司。有机磷酸酯、己二酸酯、柠檬酸酯、癸二酸酯及苯二甲酸酯类等54种替代型塑化剂标准品(具体名称见表1,纯度≥96%)购自北京曼哈格生物科技有限公司及广州佳途科技股份有限公司;芝麻油80批次:市售。Cleanert PSA/Silica固相萃取柱(规格1 g/6 mL,天津博纳艾杰尔科技有限公司)。

**表 1:** 54种替代型塑化剂的CAS号、分子式、保留时间及碎片离子精确质量数、筛查限(SDL)及定量限(LOQ)

No.	Compound	CAS number	Molecular formula	Retention time/min	Quantitative ion (m/z)	Qualitative ions (m/z)	SDL/ (mg/kg)	LOQ/ (mg/kg)
1	trimethyl phosphate (磷酸三甲酯)	512-56-1	C_3_H_9_O_4_P	3.768	110.0127	78.9943, 80.0022, 109.0049	0.01	0.02
2	triethyl phosphate (磷酸三乙酯)	78-40-0	C_6_H_15_O_4_P	5.348	155.0468	98.9842, 109.0049, 127.0155	0.01	0.02
3	diethyl maleate (马来酸二乙酯)	141-05-9	C_8_H_12_O_4_	5.713	99.0077	126.0311, 127.0390, 143.0339	0.02	0.04
4	2-ethylhexyl acrylate (丙烯酸-2-乙基己酯)	103-11-7	C_11_H_20_O_2_	6.177	70.0777	55.0178, 55.0542, 83.0855	0.02	0.04
5	dimethyl adipate (己二酸二甲酯)	627-93-0	C_8_H_14_O_4_	6.262	111.0441	101.0597, 114.0675, 143.0703	0.01	0.02
6	diethyl adipate (己二酸二乙酯)	141-28-6	C_22_H_42_O_4_	7.258	111.0441	115.0754, 128.0832, 157.0859	0.02	0.04
7	tripropyl phosphate (磷酸三丙酯)	513-08-6	C_9_H_21_O_4_P	7.276	98.9842	123.0206, 124.9998, 141.0311	0.01	0.02
8	diisopropyl adipate (己二酸二异丙酯)	6938-94-9	C_10_H_18_O_4_	7.694	129.0546	100.0519, 101.0597, 111.0441	0.01	0.02
9	trimethyl citrate (柠檬酸三甲酯)	1587-20-8	C_9_H_14_O_7_	7.830	143.0339	101.0233, 144.0373, 153.0182	0.01	0.02
10	diisobutyl fumarate (富马酸二异丁酯)	7283-69-4	C_12_H_20_O_4_	7.969	155.0703	99.0077, 147.0652, 221.0808	0.01	0.02
11	dimethyl terephthalate (对苯二甲酸二甲酯)	120-61-6	C_10_H_10_O_4_	8.106	163.0390	135.0441, 164.0424, 194.0574	0.01	0.02
12	triisobutyl phosphate (磷酸三叔丁酯)	126-71-6	C_12_H_27_O_4_P	8.128	98.9842	111.9920, 139.0155, 155.0468	0.01	0.02
13	dimethyl isophthalate (间苯二甲酸二甲酯)	1459-93-4	C_10_H_10_O_4_	8.177	163.0390	135.0441, 164.0424, 194.0574	0.01	0.02
14	dibutyl maleate (马来酸二丁酯)	105-76-0	C_12_H_20_O_4_	8.258	99.0077	57.0699, 117.0182, 155.0703	0.01	0.02
15	dimethyl azelate (壬二酸二甲酯)	1732-10-1	C_11_H_20_O_4_	8.314	152.0832	111.0804, 143.1067, 185.1172	0.02	0.04
16	dibutyl fumarate (富马酸二丁酯)	105-75-9	C_12_H_20_O_4_	8.522	155.0703	99.0077, 117.0182, 173.0808	0.01	0.02
17	dibutyl itaconate (衣康酸二丁酯)	2155-60-4	C_13_H_22_O_4_	8.619	113.0233	85.0284, 86.0362, 131.0339	0.01	0.02
18	2,2,4-trimethyl-1,3-pentanediol diisobutyrate (2,2,4-三甲基-1,3- 戊二醇二异丁酸酯)	6846-50-0	C_16_H_30_O_4_	8.678	71.0491	111.1168, 173.1172, 243.1591	0.01	0.02
19	tributyl phosphate (磷酸三丁酯)	126-73-8	C_12_H_27_O_4_P	8.929	98.9842	124.9998, 155.0468, 211.1094	0.01	0.02
20	triethyl citrate (柠檬酸三乙酯)	77-93-0	C_12_H_20_O_7_	9.003	157.0495	115.0390, 129.0182, 139.0026	0.01	0.02
21	diethyl terephthalate (对苯二甲酸二乙酯)	636-09-9	C_12_H_14_O_4_	9.024	177.0546	149.0233, 166.0261, 194.0574	0.01	0.02
22	diethyl azelate (壬二酸二乙酯)	624-17-9	C_13_H_24_O_4_	9.131	199.1329	111.0804, 152.0832, 157.1223	0.01	0.02
23	diisobutyl adipate (己二酸二异丁酯)	141-04-8	C_12_H_22_O_4_	9.143	129.0546	101.0597, 111.0441, 185.1172	0.01	0.02
24	acetyl triethyl citrate (乙酰柠檬酸三乙酯)	77-89-4	C_14_H_22_O_8_	9.542	157.0495	139.0026, 203.0914, 213.0757	0.01	0.02
25	dibutyl adipate (己二酸二丁酯)	105-99-7	C_14_H_26_O_4_	9.626	185.1172	111.0441, 129.0546, 156.1145	0.01	0.02
26	tris(2-chloroethyl) phosphate (磷酸三(2-氯乙基)酯)	115-96-8	C_6_H_12_Cl_3_O_4_P	9.676	248.9845	204.9583, 222.9688, 250.9816	0.01	0.02
27	diethyl sebacate (癸二酸二乙酯)	110-40-7	C_14_H_26_O_4_	9.75	213.1485	125.0961, 166.0988, 171.1380	0.01	0.02
28	diallyl isophthalate (间苯二甲酸二烯丙酯)	1087-21-4	C_14_H_14_O_4_	10.071	189.0546	115.0542, 149.0233, 190.0580	0.02	0.04
29	diallyl terephthalate (对苯二甲酸二烯丙酯)	1026-92-2	C_14_H_14_O_4_	10.125	189.0546	115.0542, 149.0233, 190.0580	0.02	0.04
30	tripentyl phosphate (磷酸三正戊酯)	2528-38-3	C_15_H_33_O_4_P	10.810	98.9842	124.9998, 169.0624, 239.1407	0.01	0.02
31	diisobutyl terephthalate (对苯二甲酸二异丁酯)	18699-48-4	C_16_H_22_O_4_	11.286	205.0859	149.0233, 167.0339, 223.0965	0.01	0.02
32	bis(2-ethylhexyl) maleate (马来酸二乙基己酯)	142-16-5	C_20_H_36_O_4_	12.892	117.0182	70.0777, 99.0077, 100.0155	0.01	0.02
33	dibutyl sebacate (癸二酸二正丁酯)	109-43-3	C_18_H_34_O_4_	13.062	185.1172	139.1117, 143.1067, 241.1798	0.02	0.04
34	tributyl citrate (柠檬酸三丁酯)	77-94-1	C_18_H_32_O_7_	13.324	185.0808	111.0077, 129.0182, 139.0026	0.01	0.02
35	bis(2-ethylhexyl) methylenesucci- nate (衣康酸二(2-乙基己基)酯)	2287-83-4	C_21_H_38_O_4_	13.501	113.0233	70.0777, 71.0855, 131.0339	0.02	0.04
36	acetyl tributyl citrate (乙酰柠檬酸三丁酯)	77-90-7	C_20_H_34_O_8_	14.107	185.0808	129.0182, 139.0026, 157.0132	0.01	0.02
37	bis(2-butoxyethyl) adipate (己二酸双(2-丁氧基乙基)酯)	141-18-4	C_18_H_34_O_6_	14.394	155.0703	85.0648, 111.0441, 173.0808	0.01	0.02
38	tris(1,3-dichloro-2-propyl)phos- phate (磷酸三(1,3-二氯-2-丙基)酯)	13674-87-8	C_9_H_15_Cl_6_O_4_P	15.0913	190.9426	98.9842, 192.9397, 380.8960	0.01	0.02
39	bis(2-ethylhexyl) adipate (己二酸二(2-乙基己基)酯)	103-23-1	C_22_H_42_O_4_	15.713	129.0546	70.0777, 111.0441, 207.0321	0.02	0.04
40	tris(2-butoxyethyl) phosphate (磷酸三(2-丁氧基乙基)酯)	78-51-3	C_18_H_39_O_7_P	15.903	124.9998	199.0730, 227.1043, 299.1618	0.01	0.02
41	triphenyl phosphate (磷酸三苯酯)	115-86-6	C_18_H_15_O_4_P	15.913	326.0703	215.0257, 325.0624, 327.0736	0.01	0.02
42	2-ethylhexyl diphenyl phosphate (磷酸二苯异辛酯)	1241-94-7	C_20_H_27_O_4_P	16.221	251.0468	249.0311, 250.0389, 252.0502	0.01	0.02
43	bis(1-butylpentyl) adipate (己二酸二(1-丁基戊基)酯)	77916-77-9	C_24_H_46_O_4_	16.330	129.0546	101.0597, 111.0441, 143.1430	0.01	0.02
44	tris(2-ethylhexyl) phosphate (磷酸三辛酯)	78-42-2	C_24_H_51_O_4_P	16.648	98.9842	55.0542, 70.0777, 113.1325	0.02	0.04
45	tri-o-cresyl phosphate (磷酸三邻甲苯酯)	78-30-8	C_21_H_21_O_4_P	18.307	368.1172	165.0699, 181.1012, 277.0624	0.01	0.02
46	tri-m-cresyl phosphate (磷酸三间甲苯酯)	563-04-2	C_21_H_21_O_4_P	19.074	368.1172	261.0675, 367.1094, 369.1206	0.01	0.02
47	bis(2-ethylhexyl) isophthalate (间苯二甲酸二(2-乙基)己基酯)	137-89-3	C_14_H_26_O_4_	19.398	261.1485	149.0233, 167.0339, 279.1591	0.01	0.02
48	bis(2-ethylhexyl) azelate (壬二酸二(2-乙基己基)酯)	103-24-2	C_25_H_48_O_4_	19.654	171.1016	70.0777, 152.0832, 172.1050	0.02	0.04
49	diphenyl isophthalate (间苯二甲酸二苯酯)	744-45-6	C_20_H_14_O_4_	19.867	225.0546	104.0257, 141.0699, 226.0580	0.01	0.02
50	diphenyl terephthalate (对苯二甲酸二苯酯)	1539-04-4	C_20_H_14_O_4_	19.945	225.0546	104.0257, 197.0597, 226.0580	0.01	0.02
51	tri-p-cresyl phosphate (磷酸三对甲苯酯)	78-32-0	C_21_H_21_O_4_P	20.258	368.1172	261.0675, 367.1094, 369.1206	0.02	0.04
52	bis(2-ethylhexyl) sebacate (癸二酸二(2-乙基己基)酯)	122-62-3	C_26_H_50_O_4_	20.961	185.1172	139.1117, 186.1206, 203.1278	0.02	0.04
53	dioctyl benzene-1,3-dicarboxylate (间苯二甲酸二正辛酯)	4654-18-6	C_24_H_38_O_4_	21.484	167.0339	149.0233, 261.1485, 279.1591	0.01	0.02
54	butyryl trihexyl citrate (乙酰柠檬酸三正己酯)	82469-79-2	C_26_H_46_O_8_	22.613	213.1121	129.0182, 157.0132, 315.2166	0.01	0.02

### 1.2 标准溶液的配制

准确称取各类APs标准品10 mg至10 mL容量瓶中,用正己烷溶解并定容至刻度,分别配制成质量浓度为1000 mg/L的标准储备液,置于4 ℃冰箱中保存。

移取各标准储备溶液,用正己烷分别稀释成质量浓度为0.01、0.02、0.05、0.1、0.2 mg/L的系列混合标准工作溶液。

### 1.3 样品前处理

称取0.5 g(精确至0.0001 g)试样于10 mL具塞磨口离心管中,加入3 mL乙腈,涡旋1 min,超声提取20 min, 4000 r/min离心5 min,收集上清液。残渣中加入3 mL乙腈,涡旋1 min, 4000 r/min离心5 min。合并2次上清液,待SPE净化。

SPE净化:依次加入5 mL二氯甲烷、5 mL乙腈活化,弃去流出液;将待净化液加入SPE小柱,收集流出液;再加入5 mL乙腈洗脱,收集流出液,合并两次收集的流出液,40 ℃下氮吹至近干,用正己烷准确定容至1 mL,涡旋混匀,供GC-Q-TOF/MS分析。

基质匹配工作溶液:选取空白样品,按上述方法处理至氮吹浓缩近干,分别加入1 mL 1. 2节配制的系列标准工作溶液,配制成基质匹配工作溶液,临用时现配。

### 1.4 仪器条件

#### 1.4.1 气相色谱条件

色谱柱:HP-5MS毛细管柱(固定相为5%苯基+95%聚二甲基硅氧烷)(30 m×250 μm×0.25 μm);进样口温度:280 ℃;进样模式:不分流;进样量:1 μL;载气:氦气,流速1 mL/min;程序温度:60 ℃保持1 min, 20 ℃/min升温至220 ℃保持1 min,再以5 ℃/min升温至280 ℃保持4 min。

#### 1.4.2 质谱条件

离子化模式:电子轰击离子源(EI, 70 eV);离子源温度:280 ℃;四极杆温度:150 ℃;传输线温度:290 ℃;溶剂延迟:3.5 min。采集模式:全扫描模式,扫描范围为*m/z* 50~500,扫描速率为5 spectrum/s。

### 1.5 数据库的建立

将质量浓度为0.1 mg/L的54种APs标准溶液注入仪器中,按1.4节的条件进行分析。通过对标准物质溶液进行全扫描,利用Qualitative Analysis 10.0将获得的质谱图扣除背景后与NIST标准谱库进行比较,通过碎片离子*m/z*及其分子式,进一步确定目标化合物及其保留时间和主要碎片离子等必要信息,并将质谱图发送至MassHunter PCDL Manager(B.08.00)从而构建包含有机磷酸酯、己二酸酯、柠檬酸酯、癸二酸酯及苯二甲酸酯类化合物等54种重点关注污染物的高分辨数据库。其保留时间、定量离子、定性离子信息详情见表1。

### 1.6 基质效应(ME)评价方式

本文采用ME=[基质匹配标准曲线的斜率/溶剂标准曲线的斜率-1]×100%来定量评价APs的基质效应。ME>0,表现为基质增强效应,ME<0,表现为基质抑制效应,当0≤|ME|≤20%时,说明基质效应不明显;当20%<|ME|<50%时,表现为中等强度的基质干扰,而当|ME|≥50%,则为强基质干扰。

## 2 结果与讨论

### 2.1 提取溶剂的选择

APs包括有机磷酸酯、己二酸酯、柠檬酸酯、癸二酸酯及苯二甲酸酯等类化合物,其种类繁多且性质差异较大,因此选择适当的提取溶剂尤为重要。在APs检测中常用的提取溶剂有正己烷、乙腈和甲醇等,本研究对这3种提取溶剂进行了筛选。实验结果表明,使用正己烷作为提取溶剂时会与芝麻油样品发生互溶现象,芝麻油中的脂肪会对色谱系统造成严重污染,并影响测定结果的准确性,因此不适合作为芝麻油样品的提取溶剂。基于以上考虑,在后续实验中仅考察乙腈和甲醇作为提取溶剂对芝麻油加标样品的提取效果。

本实验选取空白芝麻油样品,并添加0.1 mg/kg的54种APs标准溶液,按照1.3节步骤进行提取,然后使用GC-Q-TOF/MS对乙腈提取液和甲醇提取液进行全扫描分析。从图1a可以观察到,当甲醇作为提取溶剂时,色谱图基线较高,可能是由于甲醇具有较高极性导致共萃物较多;而乙腈提取液的全扫描色谱图相对干净且基线平稳,大多数APs显示出更高的灵敏度和分辨率。此外,在本实验中还考察了两种提取溶剂的提取效果。结果显示,在使用乙腈和甲醇作为提取溶剂时,54种APs的回收率分别在66.7%~152.1%和56.9%~219.0%范围内波动。尤其是在甲醇提取时各目标物回收率范围存在较大波动,其中部分化合物如有机磷酸酯、柠檬酸酯类表现出偏高的回收率(见图1b)。而在乙腈提取条件下,83%的化合物回收率为60%~120%。这说明乙腈具有良好的溶解性和渗透力,并适用于广泛极性范围内APs的有效提取。因此,本实验选择乙腈作为最佳的提取溶剂。

**图1:**
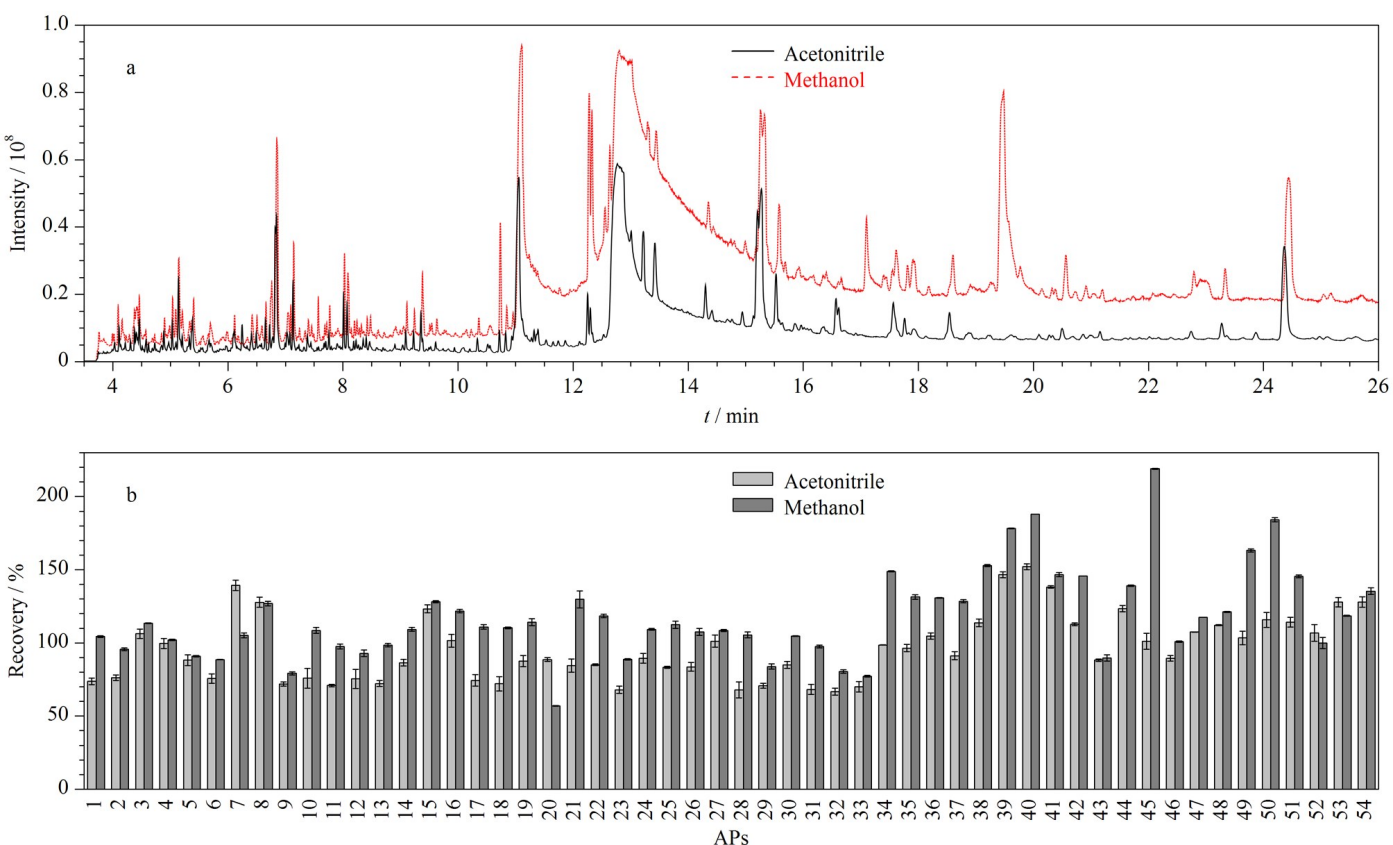
(a)甲醇和乙腈作为提取溶剂时样品的总离子流色谱图及(b)54种APs的回收率(*n*=6)

### 2.2 净化方式的选择

芝麻油含有脂肪、色素和甾醇等杂质。经乙腈提取后,具有较高lg*K*_ow_值的弱极性APs更容易被分配到脂肪中,其提取效率随着APs极性减小而降低。这说明脂肪对于低极性APs的提取效率影响很大。如果不进行净化处理直接进样,则可能导致假阳性或假阴性结果,并且会对仪器造成污染。因此,在确保54种具有较大理化差异的APs高效提取的基础上,如何有效去除油脂成为检测过程中的难点。

目前油脂样品的净化多采用凝胶渗透色谱法(GPC)、冷冻除脂、固相萃取法,其中GPC虽然可以实现自动化,但需要昂贵的仪器,检测成本高。因此,实验考察了冷冻除脂、PSA/Silica复合固相萃取柱净化两种净化方式对样品提取液的净化效果。将两种净化液与不净化样品提取液通过GC-Q-TOF/MS进行全扫描,其总离子流色谱图见图2。

**图2:**
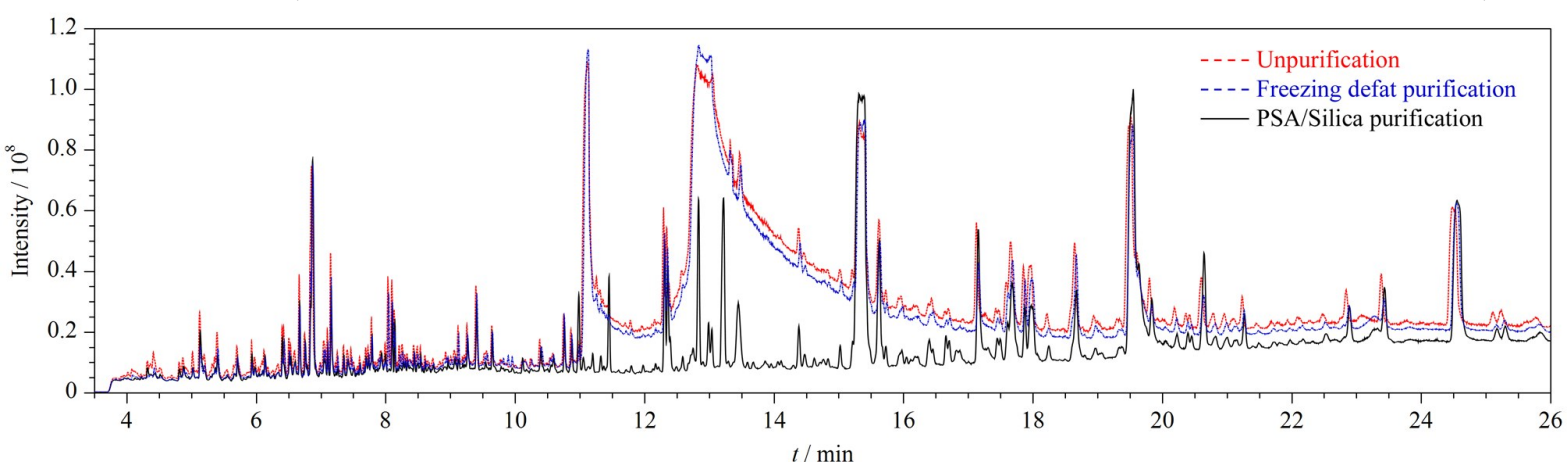
不同净化方式下样品的总离子流色谱图

冷冻除脂虽然操作简便、成本低,但该方法色谱峰基线较高、基质去除效果差,会影响定性结果的准确鉴定。而采用PSA/Silica复合固相萃取柱净化,该柱以PSA和未键合硅胶为吸附剂,可通过弱阴离子交换或极性作用有效去除芝麻油中的脂肪酸、有机酸和极性色素等大部分基质干扰,其色谱峰基线较平稳、杂质峰较少、净化效果更好;且净化柱为玻璃材质,克服了净化柱本身可能携带的微量APs造成的背景污染,能有效降低方法的空白本底值,适于大批量样品的筛查。本实验同时考察了PSA/Silica固相萃取柱净化对54种APs加标回收率的影响,结果显示,其回收率范围为68.5%~101.3%,符合检测要求。故本研究选择PSA/Silica柱固相萃取作为净化方式。

### 2.3 气相色谱条件的优化

尽管飞行时间质谱定性能力强大,具有较高的分辨率和质量精度,但由于APs种类繁多、性质相似,其同分异构体的色谱保留行为和质谱图都很相似,很容易导致假阳性问题的产生。利用气相色谱实现其同分异构体的分离非常重要,而程序升温是影响APs同分异构体分离的关键因素。因此,为了实现54种APs的有效分离,本研究通过改变气相色谱的升温程序,使用两个升温梯度,成功实现了54种APs及同分异构体的色谱分离,解决了同分异构体由于色谱无法分离而不能准确定性和定量分析的问题。在优化条件下,54种APs的总离子流图见图3。

**图3:**
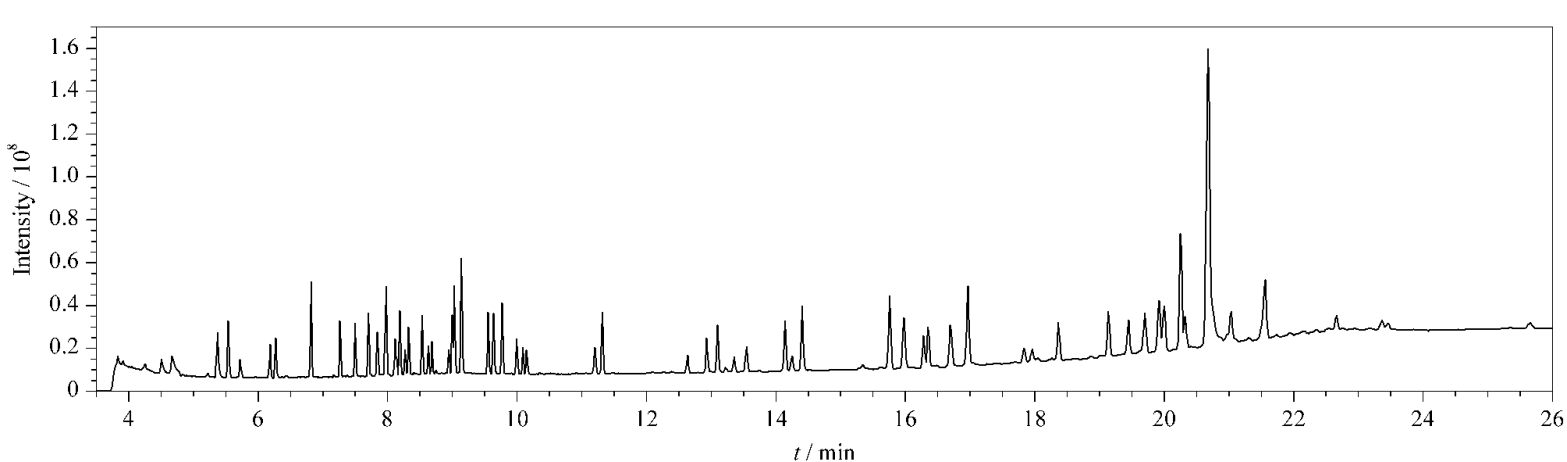
54种APs的总离子流色谱图

### 2.4 定性方法的建立

本研究结合Quantitative Analysis软件和建立的PCDL数据库进行目标物的筛查分析,为降低假阳性和假阴性分析结果的概率,需要对筛查参数进行优化,这些参数包括精确质量提取窗口、保留时间偏差等。本研究在空白芝麻油样品基质中添加0.02 mg/L的54种APs混合标准工作溶液,来优化质量提取窗口、保留时间偏差等筛查参数。

精确的质量提取窗口可以去除干扰离子、提高分析的专属性和灵敏度,使定性、定量结果更加准确。本研究将质量提取窗口分别设置为±5×10^-6^、±10×10^-6^、±15×10^-6^来考察筛查结果的准确性,结果表明,当质量提取窗口为±5×10^-6^时,有3种化合物未被筛查出来,假阴性率为5.6%,当质量提取窗口为±10×10^-6^、±15×10^-6^时,54种APs均得到鉴定,因此将质量提取窗口设置为±10×10^-6^。

保留时间偏差是另一个重要的筛查参数,设置过宽或过窄都可能会造成假阳性或假阴性。在筛查方法建立的过程中发现,色谱系统稳定性很高,保留时间偏差一般不超过0.10 min,但基质会影响部分目标物的保留时间。本研究将保留时间偏差分别设置为±0.10、±0.15、±0.20 min来进行筛查,结果显示,当保留时间偏差为±0.10 min时,有5种化合物未被筛查出来,假阴性率为9.3%,当保留时间偏差为±0.15 min和±0.20 min时,54种APs均得到鉴定,因此将保留时间偏差设置为±0.15 min。

为提高定性筛查的准确性,还对特征离子数量等其他筛查参数做了设置,每种化合物自动提取4个离子,要求其中至少两个离子检出。得到鉴定的化合物必须满足以下条件:谱库匹配综合得分>75分,至少两个定性离子检出且质量精度偏差不超过5×10^-6^,其色谱峰的信噪比≥3,碎片离子的提取色谱图保留时间在±0.10 min内,样品与标准溶液中离子相对丰度的偏差不超过30%,以上条件均满足视为阳性检出。在软件给出筛查结果后,还需手动对可疑目标物进行人工鉴定和确证。图4为芝麻油阳性样品中得到鉴定的5种替代型塑化剂的色谱图和质谱图,可以看出鉴定到的化合物有4个离子检出且质量精度偏差均在±5×10^-6^范围内。

**图4:**
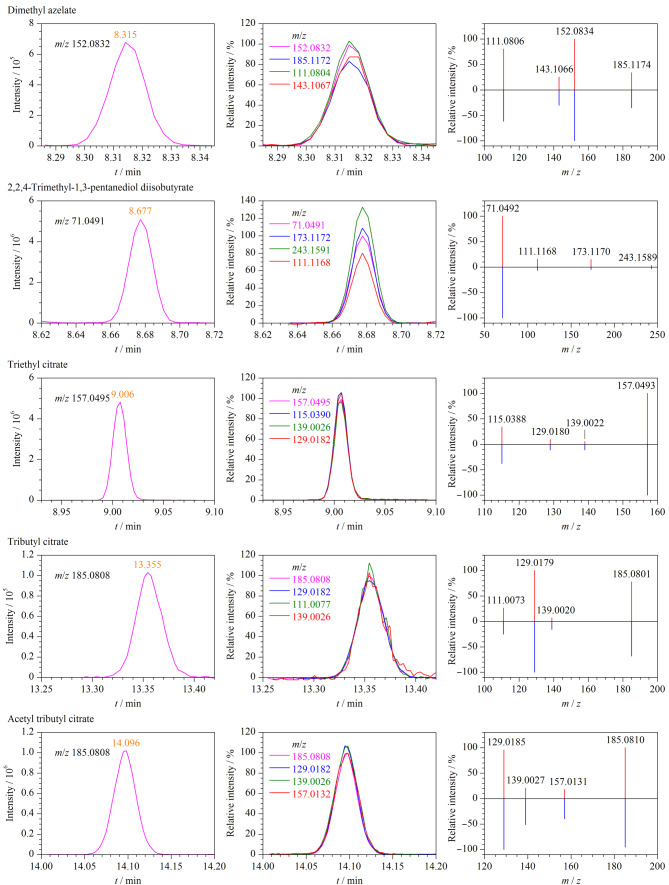
芝麻油阳性样品中5种替代型塑化剂的鉴定

### 2.5 定量方法学考察

#### 2.5.1 标准曲线

对芝麻油阳性样品中鉴定到的5种目标物进行方法学考察。基质效应实验结果显示5种目标物均存在基质抑制效应,因此,本研究采用基质匹配标准曲线法定量。在空白基质中分别加入0.01、0.02、0.05、0.1、0.2 mg/L 5种APs混合标准工作溶液,按照1.4节进行分析,以目标化合物的质量浓度为横坐标(*x*, mg/L),峰面积为纵坐标(*y*)绘制标准曲线。结果表明,在0.01~0.2 mg/L范围内5种APs线性关系良好,相关系数均大于0.99,见表2。

**表 2:** 5种替代型塑化剂线性方程、相关系数、基质效应及加标回收率(*n*=6)

No.	Compound	Linear equation	Correlation coefficient	ME/ %	0.04 mg/kg			0.08 mg/kg			0.2 mg/kg	
					Recovery/ %	RSD/ %		Recovery/ %	RSD/ %		Recovery/ %	RSD/ %
1	dimethyl azelate	y=2037312x+3291	0.9973	-16.2	82.0	2.5		78.1	4.3		81.7	1.3
2	2,2,4-trimethyl-1,3-pentanediol diisobutyrate	y=6346831x+702936	0.9981	-25.7	94.0	5.6		83.0	6.1		79.8	2.8
3	triethyl citrate	y=17014666x+32188	0.9954	-21.6	85.4	0.4		74.5	2.5		71.3	4.0
4	tributyl citrate	y=5262182x-8865	0.9998	-4.9	94.1	1.9		85.5	3.0		84.7	3.4
5	acetyl tributyl citrate	y=15467371x+61056	0.9975	-22.0	97.8	1.0		83.2	1.1		86.6	3.2

*y*: peak area; *x*: mass concentration, mg/L.

#### 2.5.2 筛查限与定量限

SDL是参照欧盟健康和食品安全总司SANTE/11312/2021指导文件的要求,选取空白芝麻油样品,在0.01、0.02、0.04 mg/kg 3个不同添加水平下,每个水平重复测定20次,在某个水平下有95%以上样品能被筛查出,即假阴性率≤5%时则认为该水平为此化合物的筛查限。定量限是通过向空白芝麻油样品中添加不同量的混合标准工作溶液,以信噪比*S/N*≥10对应的添加量作为目标物的定量限。54种APs的筛查限及定量限结果见表1。结果表明,54种APs的SDL为0.01~0.02 mg/kg,其中41种化合物的筛查限为0.01 mg/kg, 13种化合物的筛查限为0.02 mg/kg,定量限为0.02~0.04 mg/kg,这表明构建的筛查方法对54种APs具有良好的灵敏度。方法假阴性率低于5%,满足SANTE/11312/2021指导文件对筛查结果的要求。

#### 2.5.3 准确度和精密度

采用空白基质加标,分别在0.04、0.08、0.2 mg/kg 3个加标水平下同时测定6次,对5种APs进行准确度和精密度试验,其回收率为71.3%~97.8%,相对标准偏差(RSD)为0.4%~6.1%,这表明构建的靶向筛查定量方法具有良好的准确度及精密度。

### 2.6 实际样品的测定

为验证该方法的可靠性,对市售的80批次芝麻油进行筛查,结果表明有5批次样品检出5种APs。这5种目标物分别为柠檬酸三乙酯(TEC)、柠檬酸三丁酯、ATBC、壬二酸二甲酯和TXIB,其检出值分别为0.0736、0.0552、0.0604、0.2250、0.0732 mg/kg。其中,TEC为欧盟(EU)No 10/2011允许使用的食品接触材料添加剂,ATBC、TXIB均为我国GB 9685-2016允许用于食品接触材料的添加剂,柠檬酸三丁酯和壬二酸二甲酯为美国食品药品监督管理局(FDA)授权使用的食品接触物质间接添加剂,除规定TXIB在食品中的SML为5 mg/kg外,其他物质在食品中的SML(T)为60 mg/kg,样品检出值均符合国内外标准及法规的限量要求。

### 2.7 可回溯性分析

GC-Q-TOF/MS采集全谱数据,数据采集与PCDL库中的目标物数目无关,因此可实现回溯性分析,即数据采集完成后仍可对数据进行重新分析,可扩大目标物范围。比如筛查1种新的APs对苯二甲酸二(2-乙基)己酯,只需将它的保留时间、分子式、精确分子质量数和CAS号等信息发送到54种APs的PCDL库中,按照2.4节选定的筛查参数进行筛查即可。回溯性分析可实现目标化合物的实时扩充,不需要重新采集数据便可实现样品中目标物的高通量筛查和定量分析,是未来污染物风险监测技术的发展方向。

## 3 结论

本研究建立了芝麻油中54种APs的快速筛查策略,利用GC-Q-TOF/MS建立的PCDL高分辨质谱数据库结合定量分析软件可同时对芝麻油中54种APs进行广谱筛查、定性鉴别及5种阳性样品的定量分析。目标物鉴定分析结合精确质量数提取窗口、保留时间偏差等参数进行定性分析,避免了假阳性和假阴性结果的出现,大大提高了检测的准确度;经GC-Q-TOF/MS采集的全谱数据可进行回溯性分析,PCDL数据库可实现目标物的实时扩充与完善,大大拓展了APs的筛查分析范围。相比于气相色谱-串联质谱方法,该筛查策略具有快速、简便、高效、准确等优势,具有很好的实际应用前景,可广泛应用于芝麻油中APs类风险物质的高通量筛查,为食品安全风险评估和风险监测提供有力的技术支撑,并为其他类别污染物的精准检测提供了解决方案。
